# The study of angiogenesis in early rheumatoid arthritis – clinical, immunohistochemical and immunological correlations

**Published:** 2008-08-15

**Authors:** Rosu A, Ciurea P, Simionescu C, Margaritescu C, Musetescu A. E., Ciurea R, Vreju A.F.

**Affiliations:** * Department of Rheumatology, University of Medicine and Pharmacy Craiova; ** Department of Pathology, University of Medicine and Pharmacy Craiova

**Keywords:** early rheumatoid arthritis, angiogenesis

## Abstract

**Objective:** The objective of the study was to analyze several immunohistochemical, histological, and morphometrical aspects of angiogenesis in early rheumatoid arthritis synovium. We aimed to identify possible correlations between the histological and immunohistochemical patterns and the serum levels of VEGF, as well as with clinical and biological markers of disease activity.

**Methods:** 35 patients with early rheumatoid arthritis – below 12 months from the onset, naive for DMARDs, underwent clinical standard examination as well as serum determinations for CRP, RF, anti-CCP2 antibodies and VEGF. DAS28 value has been determined for each patient in order to assess the disease activity. We performed biopsy sampling through arthroscopy, the synovium fragments beeing histopathologically processed, in order to elaborate a total histological score. Immunohistochemical analysis has been performed with quantification of synovial VEGF, VEGF-R1 and CD34 expression. Standard and activated microvascular density (sMVD and aMVD) have been evaluated through double immunostaining (CD34/ VEGF-R1).

**Results:** VEGF and VEGF-R1 have been identified with high prevalence in endothelial cells, in lining and sublining synovial cells, as well as in inflammatory cells. The study focuses on the analysis of aMVD, a valuable parameter, representative for active angiogenesis, which proved to correlate significantly with the serum levels of VEGF, the composite histological score as well as with VEGF-R1 and DAS28.

**Conclusion:** The statistic analysis of the data support VEGF-R1 and aMVD as markers with predictive value regarding activity and progression in early stages of rheumatoid arthritis. The validation of preliminary conclusions oblige to continuous research through extending the study group and inclusion of several others biomarkers involved in synovial angiogenesis.

## Background

Rheumatoid arthritis is characterized by the attack of the extensive pannus over the chondral structures and subchondral bone, with subsequent formation of erosive lesions. Angiogenesis represents an essential event in promoting the rheumatoid synovitis, the expansive vascular wave, offering the necessary blood supply for the cartilage nutrition as well as the access of the cellular populations with proinflammatory functions [**1**, **2**]. This way, neoangiogenesis represents sincrone phenomena with the pannus progression [**3**]. The access to the synovial handling – easier, minimum invasive as well as guided to the target areas – gave the opportunity in the last years to a deep approach of the dynamics of angiogenetic events from the inaugural stages of rheumatoid arthritis [**4**,**5**]. Several histopathological patterns of rheumatoid synovium have been described [**6**, **7**].

Each type of histopathological pattern corresponds to a certain degree of immune activity and cytokine production, the presence of lymphoid follicles being associated with the most aggressive trend of destructive disease. Meanwhile, there is also a variability of synovial patterns in the natural history of rheumatoid arthritis [**8**, **9**] that distinguishes the morphological aspects from other rheumatic diseases [**10**].

A domain of interest in early rheumatoid arthritis is represented by angiogenesis. Several endothelial growing factors have been identified in the rheumatoid synovium, of which the most specific VEGF, induced by hypoxia, has been detected in serum, synovial fluid and rheumatoid synovium [**11**,**12**]. In the dynamics of angiogenesis, the intervention of two tyrosinekinase receptors – VEGF-R1 and VEGF-R2 [**13**, **14**] - has been identified as responsible for the growing signals transmitted to the endothelial cells [**15**]; more, VEGF-R1 blockade in animal models determined the abortion of the joint destruction process [**16**], confirming the supposition that VEGF/VEGF-R1 overexpression is a promoter of osteochondral destruction, starting from the early stages of rheumatoid arthritis [**17**].

The aim of our study was to analyze several immunohistochemical, histological and morphometrical aspects of angiogenesis in patients with early rheumatoid arthritis and to identify possible correlations with the serum levels of VEGF and with the clinical and biological markers of disease activity.

## Methods

The study group included 35 patients with early rheumatoid arthritis – below 12 months from the onset, naive for DMARDs, in whom demographic data – gender, age, disease duration, the number of tender and swollen joints, HAQ score (Health Assessment Questionnaire) and serum samples for CRP, RF, anti-CCP2 antibodies and VEGF determination were recorded.

Written, informed consent has been obtained from each patient before enrolling into the study. DAS28 value has been determined for each patient. They all had active synovitis of the knee, confirmed ultrasonographically, with the identification of the target biopsy sites.

The study was approved by the Ethics Committee of the University of Medicine and Pharmacy Craiova.

**Synovial biopsy handling**

The synovial biopsy sampling has been performed in all patients through arthroscopy using the triangulation technique, in the Department of Orthopedics and Trauma of UMF Craiova. Biopsy specimens were fixed in 4% formaldehyde and embedded in paraffin.

**Histological analysis**

The synovium fragments sampled have been subject to histopathological processing by the usual technique of paraffin wax inclusion. Evaluation of the cases by histopathological analysis has been performed on the grounds of the following parameters: 

- ***the degree of synovial cell proliferation:*** less than 3 rows (0), 3-4 rows (1), 5-6 rows (2), more than 6 rows (3); 

- ***the degree of lymphoid and plasma cell infiltrate:*** diffuse (0), aggregates of lymphoid cells (1), the presence of lymphoid follicles (2), the presence of lymphoid follicles with germinative centers (3); 

- ***fibrinoid necrosis:*** absent (0), low (1), moderate (2), severe (3);

- ***mesenchymoid transformation:*** absent (0), low (1), moderate (2), severe (3);

- ***neovascularization*** evaluated through computerized morphometry (Lucia M), after vessel staining.

The evaluation of the histological pattern has been achieved using a hybrid composite index defined as the sum of the scores corresponding to each analyzed parameter.

**Immunohistochemical analysis**

*Monostainings* (VEGF and VEGF-R1)

We performed immunostaining on formalin-fixed, paraffin embedded tissue sections using the CSA II, Biotin-Free Catalyzed Amplification System (Dako-K1497) and Monoclonal Mouse Anti-Human Vascular Endothelial Growth Factor (Dako, M7273, 1:500 dilution) and Monoclonal Anti-Mouse VEGF-R1 (Flt-1) Antibody (R&D System, MAB471, dilution 1:300). In brief, sections from each paraffin-embedded block were cut at a thickness of 4μm, deparaffinized in xylene and rehydrated through graded concentrations of alcohol. Thereafter, antigen retrieval was performed by microwave heating in Tris-EDTA (pH 9) for 20 minutes for VEGF and in Citrate Buffer (pH 6) for 20 minutes for VEGF-R1. The endogenous peroxidase activity was blocked with 3% hydrogen peroxide in water and in order to reduce the background or unspecific staining we used Normal Goat Serum Blocking Solution in BSA for 30 minutes. Further more, we pursued the steps from the Manual Staining Procedure of Dako CSA II. Diaminobezidinetetrahydrochloride (DAB) was used as a chromogen. All sections were then counterstained with hematoxylin.

Quantification of antibody expression

The VEGF expression was quantified using the following scoring method: score 1 for less than 10% lining synoviocytes, score 2 for 10-25% positive cells, score 3 for 25-50% positive cells and score 4 for more than 50% positive cells.

The VEGF-R1 expression was quantified using the following scoring method: score 1 for less than 10% of sublining synovial blood vessels (SBV), score 2 for 10-25% of SBV, score 3 for 25-50% of SBV and score 4 for more than 50% of SBV.

Double immunostaining (CD34/ VEGF-R1) for the estimation of activated micro vessel density (aMVD)

We performed a double immunostaining with Monoclonal Mouse Anti-Human CD34 (Dako, M0823, 1:50 dilution) and Monoclonal Mouse Anti- Human VEGF-R1 (Flt-1) Antibody (R&D System, MAB471, dilution 1:30), using the En Vision G2 Double stain System, Rabbit/Mouse (DAB+/Permanent Red) from Dako (K5361).

Quantification of standard MVD (sMVD) and activated MVD (aMVD)

Quantification of sMVD and aMVD were estimated with the same protocol. Sections were scanned at low power (×40 and ×100). The aMVD was assessed in all optical fields by counting vascular structures at high power magnification ×200 with clearly defined expression of CD34 and VEGF-R1. The final aMVD was the mean score obtained from three fields with the highest individual scores, in order to assess the maximum angiogenic activity for each case.

**ELISA of VEGF in serum**

Serum VEGF levels were determined at the time of arthroscopy, with a standard sandwich enzyme-linked immunoabsorbent assay (ELISA) (VEGF2, DRG International, IRC, USA) using specific monoclonal and polyclonal antibodies, according to the manufacturer’s protocols. For each analysis, 100μl of serum was used. All the analyses were performed in duplicate. Sensitivity for VEGF was 40-600pg/ml.

**Statistics**

The data were analyzed using the Microsoft Excel software. General characteristics of the study group were investigated based on numbers and percentage. The disease activity score, disease duration were analyzed using means, minimum, maximum and standard deviation. Relations among the factors were analyzed using Pearson' correlation coefficient and regression analyses.

## Results 

Demographical, clinical and biological characteristics of the study group are shown in **Table 1**. 35 consecutive early RA patients were analyzed. Disease duration (mean ± S.D.) was 6.06±2.38SD months, 28-joint disease activity score (DAS28) was 4.72±1.63 SD, 8% of patients were positive for RF and the mean values for anti-CCP2 was 102.08U ± 87.05 SD.

**Table 1:** Demographical, clinical and biological characteristics of the study group

Sex F/ M	Age (years)	Disease duration (months)	DAS28	Rheumatoid Factor (U)	antiCCP2 (U)	**serum VEGF** (pg/ml)
27/ 8	48,8± 6,29 SD	6.06± 2.38SD	4.72± 1,63 SD	23,05± 5,32SD	102,08± 87,05SD	674,94± 311,57SD

**Histopathological evaluation**

**Fig.1:**
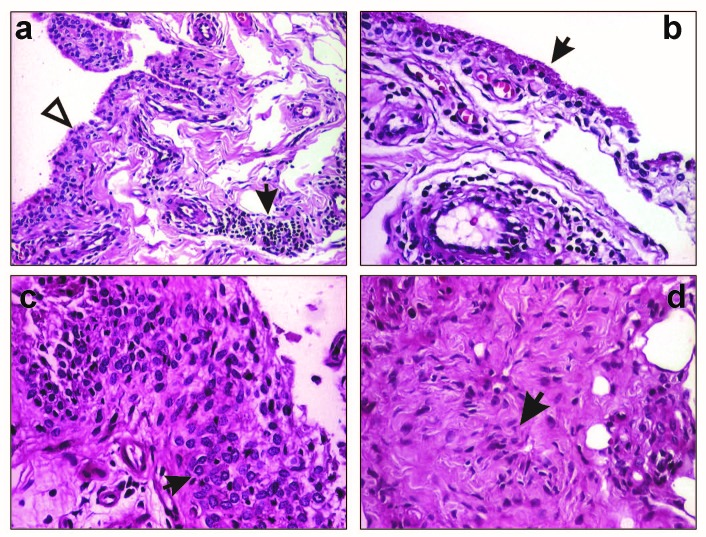
Reumathoid arthritis: a) Proliferation of lining (white triangle) and sublining synoviocytes (3-4 layers), perivascular inflammatory infiltrate (arrow head) HE staining, X100; b) Fibrinoid necrosis (arrow head) of the synoviocytes, HE staining, X200; c) Palisade of the synoviocytes - more than 6 layers (arrow head), HE stain ing, X200, d) synovial fibroblasts (arrow head), HE staining, X200.

The histopathological study concerned the magnitude of synoviocyte proliferation, of lymphoplasmocytic infiltrate, of fibrinoid necrosis and mesenchymoid transformation. (**fig. 1 a, b, c, d**).

A composite score with a maximum of 15 was recorded, with a mean of 7.17±3.58 SD (95% CI). The correlations found between the analyzed parameters have not been validated.

**Immunohistochemical evaluation**

**Fig. 2:**
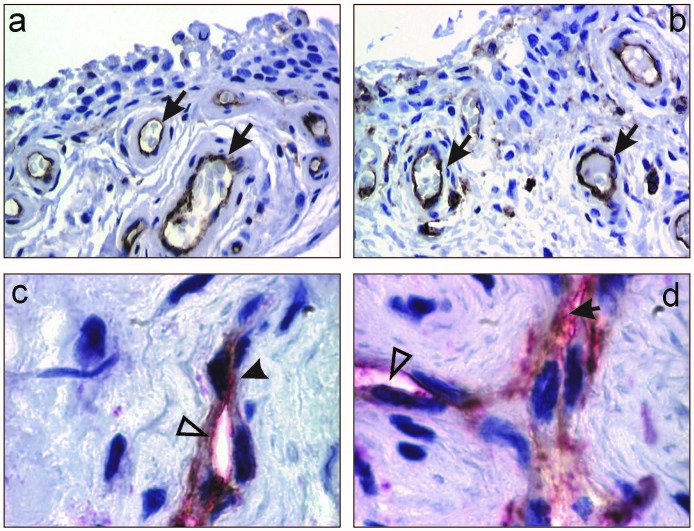
Reumathoid arthritis: a-b) CD34 (brown) positive vessels beneath the lining synovium (arrow head), X200; c-d) CD34 (brown-arrow head) and VEGF-R1(red-white triangle) positive vessels, X1000.

The study of the synovial expression of VEGF showed positivity predominantly in the lining and sublining synoviocytes and only scarcely in fibroblastic populations, inflammatory and endothelial cells (**fig. 2 a, b**). The semiquantitative evaluation of VEGF immunoexpression in synoviocytes revealed scores of 1 in 71.4% and 2 in 28.6% of cases.

VEGF-R1 expression analysis showed positivity especially at endothelial level but also in lining, sublining synoviocytes as well in inflammatory cells (**fig. 2 c, d**). Semiquantitative quantification of VEGF-R1 showed values considered as a score of 1 (31.4%), 2 (40%) and 3 (28.5%).

**Fig. 3:**
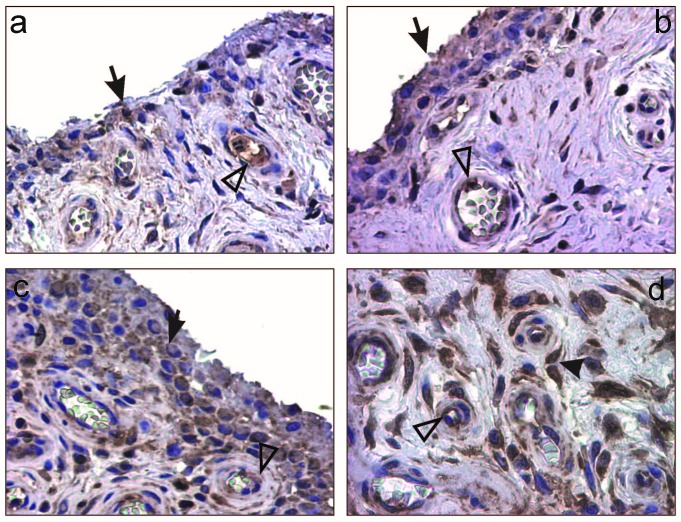
Reumathoid arthritis: a-b) VEGF positive lining synoviocytes (brown-arrow) and endothelial blood cells (brown-white triangle), X200; c-d) VEGF-R1 positive lining synoviocytes (brown-arrow), endothelial blood cells (brown-white triangle) and synovial fibroblasts (brown-arrow head), X200, X400.

Standard microvascular density (sMVD), interpreted after CD34 immunostaining for superficial subsynoviocytar located vessels (**fig. 3 a, b**) revealed a mean of 57.37/mm² ±4.85 SD (95% CI).

Activated microvascular density (aMVD) has been evaluated after double immunostaining (CD34, VEGF-R1) for the superficial subsynoviocytar vessels (**fig. 3 c, d**), with a mean value of 27.65/mm² ±5.22SD (95% CI).

**Statistical analysis**

We observed significant correlations between the serum levels of VEGF and DAS28 score (r=0.90), (**fig. 4a**), between DAS28 and the total composite histological score (r=0.90), as well as between aMVD and serum VEGF (r=0.89) (**fig. 4b**), DAS28 (r=0.88) (**fig. 4c**), composite histological score (r=0.82) and VEGF-R1 (r=0.81).

Instead, we observed only a tendency of correlation between serum and synovial VEGF levels (r=0.56) as well as synovial VEGF and DAS28 (r=0.60).

There is a significant statistical correlation between VEGF-R1 and aMVD – r= 0,81 (**fig. 4d**).

**Figure:**
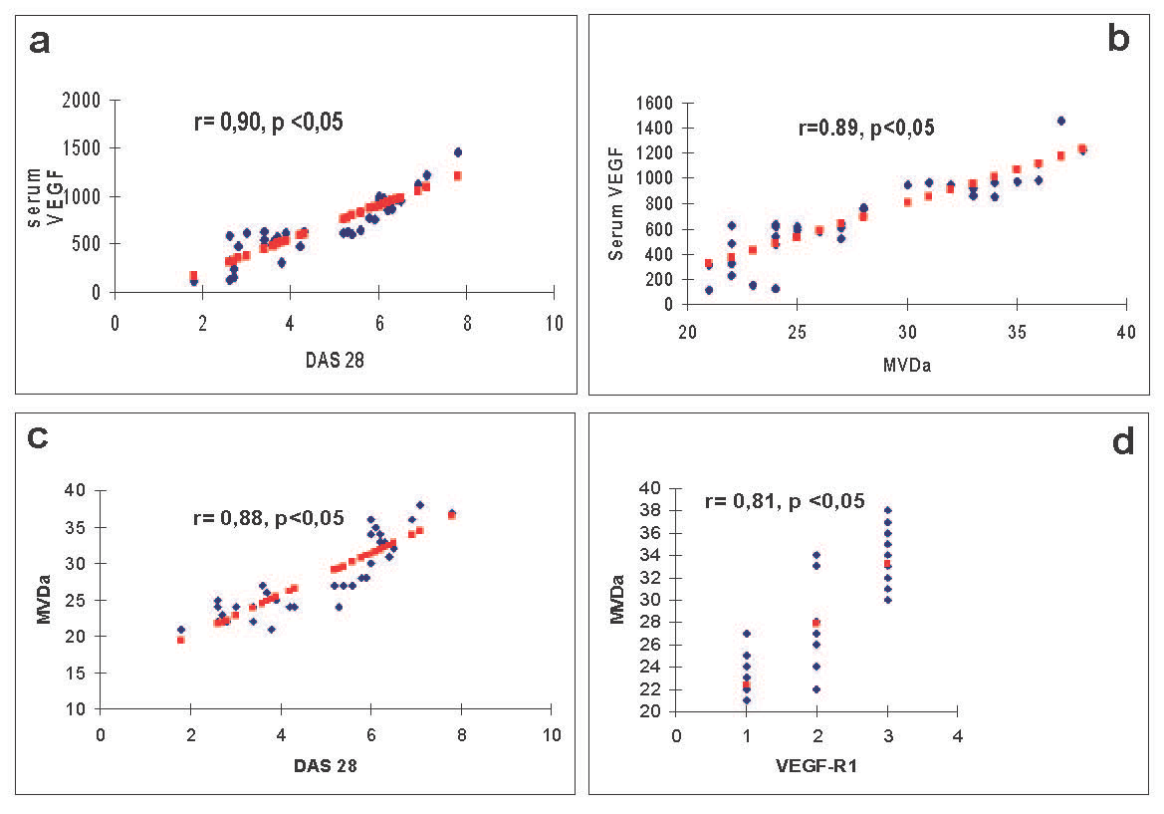


We did not identify any correlations of the analyzed parameters with gender or with the disease duration.

## Discussion

The progression of joint destructive process in rheumatoid arthritis is still unpredictable, with different evolutive patterns. Defining the severity profile in early stages of rheumatoid arthritis is a continuous concern of numerous studies [**18**, **19**, **20**], in the attempt of identifying a panel of biomarkers with predictive valences in early rheumatoid arthritis.

Angiogenesis is a complex process that amplifies and/or perpetuates synovial inflammation with redundant effects on accessing pathogenically pathways and a high vascular turnover [**21**, **22**].

Identifying and grading the histopathological patterns regarding synoviocyte proliferation, lymphoplasmocytic infiltrate, fibrinoid necrosis, mesenchymoid transformation, neovascularisation – allowed us to elaborate a scoring system [**7**, **23**] evocative for disease activity. In our study, the composite histological score has been significantly correlated with the disease activity indicated by DAS28 scoring.

VEGF represents a potent mediator of endothelial proliferation, with a critical role in promoting angiogenesis. Results from numerous studies [**6**, **12**, **24**, **25**] confirm significant correlations between the serum levels of VEGF and clinical, biological parameters of disease activity - the number of swollen joints, CRP, RF. Our results support the correlation between the serum levels of VEGF and the common validated parameter for disease activity in rheumatoid arthritis – DAS28.

Of high importance in defining the role of VEGF in synovial angiogenesis are the synovial immunoexpression of VEGF and VEGF-R1, VEGF-R2, reports of several studies [**13**-**16**, **26**,**27**] locate VEGF in perivascular cells, sublining, lining and endothelial cells, while VEGF-R1 and VEGF-R2 occupy the same synovial sites as VEGF [**15**].

In our study VEGF and VEGF-R1 have been identified with high prevalence in endothelial cells, in lining and sublining synovial cells, as well as in inflammatory cells, supporting the role of VEGF – VEGF receptor system in the management of growth signaling and vascular proliferation in rheumatoid synovium. We observed a trend of correlation between the synovial VEGF and DAS28 as well as between serum and synovial VEGF, analyzing a broader study group being relevant in defining a significant correlation between the two parameters. 

The study approached the analysis of activated microvascular density (aMVD), a parameter of activated angiogenesis [**28**] that proved to correlate significantly with the serum levels of VEGF, the composite histological score as well as with VEGF-R1 and DAS28. These statements support the hypothesis that asserts aMVD as a marker of disease activity and progression trend, starting from early stages of rheumatoid arthritis.

In summary, the results of the present study engage some of the most important markers involved in estimating the magnitude of angiogenesis in early rheumatoid arthritis.

The statistic analysis of the data support VEGF-R1 and aMVD as markers with predictive value regarding activity and progression in early forms of the disease. The validation of preliminary conclusions oblige to continuous research through extending the study group and inclusion of several others biomarkers involved in synovial angiogenesis.

**Abbreviations**

Anti-CCP2 antibodies – antibodies against cyclic citrullinated peptide; CRP – C reactive protein; DAS28 – disease activity score on 28 joints count; DMARDs – disease modifying anti-rheumatic drugs; aMVD – activated microvascular density; sMVD – standard microvascular density; RF – rheumatoid factor; VEGF – vascular endothelial growth factor; VEGF-R1, R2 - vascular endothelial growth factor receptors 1and 2.

**Acknowledgements**

The work was supported by the research grant 953/2007 from the ANCS-CNCSIS.
